# Transcranial ultrasound stimulation for post-stroke dysfunction: a meta-analysis of randomized controlled trials

**DOI:** 10.3389/fnins.2026.1828888

**Published:** 2026-08-07

**Authors:** Huiping Li, Jingming Liu, Minghui Wang, Lei Wang, Nana Luo, Wei Xu

**Affiliations:** 1 The First Affiliated Hospital of Hunan Normal University Hunan Provincial People’s Hospital, Changsha, China; 2 Department of Rehabilitation, The First Affiliated Hospital of Shaoyang University, Shaoyang, China; 3 Department of Neurology, The Affiliated Changsha Central Hospital, Hengyang Medical School, University of South China, Changsha, China

**Keywords:** meta-analysis, neurological function recovery, randomized controlled trial, rehabilitation, stroke, transcranial ultrasound stimulation

## Abstract

**Objective:**

To systematically evaluate the efficacy of transcranial ultrasound stimulation (TUS) in improving neurological deficits, motor, cognitive, swallowing function, and activities of daily living (ADL) in stroke patients

**Methods:**

PubMed, Embase, Web of Science, Cochrane Library, CNKI, and Wanfang Database were searched for randomized controlled trials (RCTs) on TUS for stroke patients from inception to December 1, 2025. Two reviewers independently conducted literature screening, data extraction, risk of bias assessment using the Cochrane tool, and methodological quality evaluation using the PEDro scale. Meta-analysis was performed with RevMan 5.3, and publication bias was assessed using Egger’s test in Stata 18.0

**Results:**

Twenty-one RCTs involving 1,748 patients were included. TUS combined with conventional rehabilitation significantly improved neurological deficits (NIHSS: MD = −1.98, 95% CI: −3.61 to −0.34, *P* = 0.02), upper-limb motor function (FMA-UE: MD = 7.96, 95% CI: 7.16–8.76, *P* < 0.001), lower-limb motor function (FMA-LE: MD = 3.98, 95% CI: 2.70–5.26, *P* < 0.001), cognitive function (MMSE: MD = 1.22, 95% CI: 0.74–1.71, *P* < 0.001), ADL (BI: MD = 9.08, 95% CI: 5.72–12.44, *P* < 0.001), and swallowing (Fujishima-Ichiro: MD = 1.72, 95% CI: 1.30–2.13, *P* < 0.001) Subgroup analyses suggested that cognitive benefits were more pronounced in non-acute patients, while ADL improvements were greater in those with moderate-to-severe dependence. Regarding TUS parameters, 800 kHz provided stable motor recovery, and unilateral temporal window stimulation was sufficient for meaningful functional gains. Methodological quality was acceptable (3 good, 18 fair trials), and no publication bias was detected for BI score (*P* > 0.05)

**Conclusion:**

Current evidence supports that TUS combined with rehabilitation effectively promotes recovery of neurological, motor, cognitive, swallowing, and daily living functions in stroke patients, with particular value for those with moderate-to-severe dysfunction.

**Systematic review registration:**

https://www.crd.york.ac.uk/prospero/, identifier CRD420261296388.

## 1 Introduction

According to the Global Burden of Disease (GBD) study, stroke remains the second leading cause of death and the third leading cause of death and disability worldwide (Feigin et al., 2025). Although age-standardized rates of incidence, mortality, and disability-adjusted life years (DALYs) have declined, the absolute numbers of incident cases, deaths, and DALYs have increased significantly since 1990. Low- and middle-income countries bear the vast majority of the disease burden (Feigin et al., 2025; He et al., 2024; Luo et al., 2024). Stroke often leads to multifaceted dysfunctions, including motor, cognitive, swallowing, and daily activity impairments. These issues severely affect patients’ quality of life and rehabilitation prognosis (Guo et al., 2022). While conventional treatments have certain efficacy, some patients still experience limited functional recovery. Therefore, seeking safe and effective therapeutic interventions has become a focus of clinical research.

Transcranial ultrasound stimulation (TUS) is a non-invasive neuromodulation technique that has shown potential value in stroke rehabilitation in recent years. It has emerged as a research hotspot due to its strong penetrability and good focality (Fomenko et al., 2018). The therapeutic effects of TUS are derived from multi-pathway, multi-target neuromodulatory mechanisms. Through mechanical and thermal effects, TUS can dilate cerebral vessels and improve local cerebral blood perfusion, while also promoting angiogenesis and the expression of neurotrophic factors, thereby creating a favorable microenvironment for neural repair (Qi et al., 2024; Wang et al., 2025; Shu et al., 2026). On the other hand, TUS can modulate ion channel activity and neurotransmitter release, regulate cortical excitability, inhibit neuroinflammation, and enhance synaptic remodeling and axonal sprouting, thereby promoting the reorganization and functional recovery of damaged neural networks (Issa et al., 2026). Additionally, TUS has been shown to induce synaptic plasticity changes resembling long-term potentiation (LTP), a process dependent on the activation of NMDA receptors and voltage-gated ion channels (Desai et al., 2025), providing a direct synaptic basis for motor skill re-learning and consolidation. These mechanisms underpin TUS’s ability to improve multifaceted deficits in stroke patients.

However, current studies evaluating TUS efficacy in stroke rehabilitation use varying parameters and show inconsistent conclusions, lacking systematic evidence integration. Thus, this study systematically searched Chinese and English databases for relevant randomized controlled trials up to December 1, 2025. A meta-analysis was conducted to comprehensively evaluate the effects of adding TUS to conventional basic treatment on neurological function, motor function, cognitive function, activities of daily living, and swallowing function in stroke patients. The aim is to provide evidence-based guidance for clinical practice and future research.

## 2 Materials and methods

### 2.1 Registration and guidelines

This study has been registered in the International Prospective Register of Systematic Reviews (PROSPERO) with registration number CRD420261296388 and strictly adheres to the Preferred Reporting Items for Systematic Reviews and Meta-Analyses (PRISMA) guidelines.

### 2.2 Inclusion and exclusion criteria

Inclusion criteria: ➀Participants: Adults diagnosed with ischemic or hemorrhagic stroke. ➁Interventions: The control group received sham stimulation and/or conventional rehabilitation therapy, such as motor therapy, physical modality therapy, occupational therapy, traditional Chinese medicine therapy, routine nursing care, or other rehabilitation therapies. The experimental group received therapeutic ultrasound stimulation (TUS), with any parameters or frequency, in addition to the same control intervention. ➂Outcomes: At least one of the following: upper extremity Fugl-Meyer Assessment (FMA) scale score, Barthel Index (BI) score, NIHSS score, or Fujishima-Ichiro Dysphagia Score. ➃Study design: RCT.

Exclusion criteria: ➀The study subjects include those with severe traumatic brain injury, advanced dementia, intracranial tumors, and other central nervous system lesions. ➁The intervention protocol involves confounding neuromodulation methods (simultaneous use of tDCS, rTMS, and other cranial nerve stimulations), making it impossible to isolate the effect of TUS alone. ➂No raw mean/standard deviation data are available for continuous outcome measures; ➃Redundant or duplicate publications.

### 2.3 Search strategy

We systematically searched PubMed, Embase, Web of Science, Cochrane Library, Wanfang Medical Network, and CNKI databases for RCTs on TUS for stroke patients from inception to December 1, 2025. The search employed a combination of MeSH terms and free-text words, adapted for each database’s specific characteristics, searching in fields including subject, title, keywords, and abstract. The search strategies for each database are detailed in Supplementary Appendix.

### 2.4 Study selection and data extraction

Literature was managed with Zotero 7.0. After removing duplicates, two reviewers independently selected studies. First, titles and abstracts were screened to remove clearly irrelevant studies; reasons for exclusion were recorded. Next, the remaining full texts were downloaded, read, and checked against inclusion and exclusion criteria; exclusion reasons were also recorded. Finally, reference lists of included studies were checked for other eligible studies. Data extraction included: ➀Basic study details such as author, year, type, disease duration, sample size, age, sex, treatment, interventions, diagnostic criteria, and TUS parameters. ➁Methods including participant allocation, blinding, and statistical analyses. ➂Outcomes including FMA scores, BI or MBI scores, NIHSS scores, and Fujishima-Ichiro Dysphagia Score. Disagreements between reviewers were resolved by discussing with a third reviewer until an agreement was reached.

### 2.5 Quality assessment of included studies

#### 2.5.1 Cochrane risk of bias assessment

The Cochrane Handbook for Systematic Reviews of Interventions was used to assess the risk of bias in the included RCTs. This tool evaluates potential bias across seven domains: ➀random sequence generation; ➁allocation concealment; ➂blinding of participants and personnel; ➃blinding of outcome assessment; ➄incomplete outcome data; ➅selective reporting; ➆other sources of bias. Each domain was judged as “low risk,” “unclear,” or “high risk.”

#### 2.5.2 PEDro scale assessment

In addition to the Cochrane tool, we also assessed the methodological quality of each included RCT using the Physiotherapy Evidence Database (PEDro) scale. The PEDro scale comprises 11 items, with item 1 (eligibility criteria) not counted toward the total score, yielding a maximum score of 10. Scores of 9–10 are considered “excellent,” 6–8 “good,” 4–5 “fair,” and below 4 “poor.” Two reviewers independently rated each study, and disagreements were resolved by consensus with a third reviewer.

### 2.6 Statistical analysis

We performed the meta-analysis using RevMan 5.3. We chose effect measures by variable type: mean difference (MD) with 95% CI for continuous variables and odds ratio (OR) with 95% CI for dichotomous variables. Forest plots showed the pooled effect sizes. We assessed heterogeneity with the *I*^2^ statistic. If *I*^2^ was ≤ 50%, we used a fixed-effect model; if *I*^2^ was > 50%, we used a random-effects model. We performed sensitivity analysis by omitting individual studies one at a time. To assess publication bias, we used funnel plots and Egger’s test, with α = 0.05 as the threshold for significance.

## 3 Results

### 3.1 Literature search results

**FIGURE 1:**
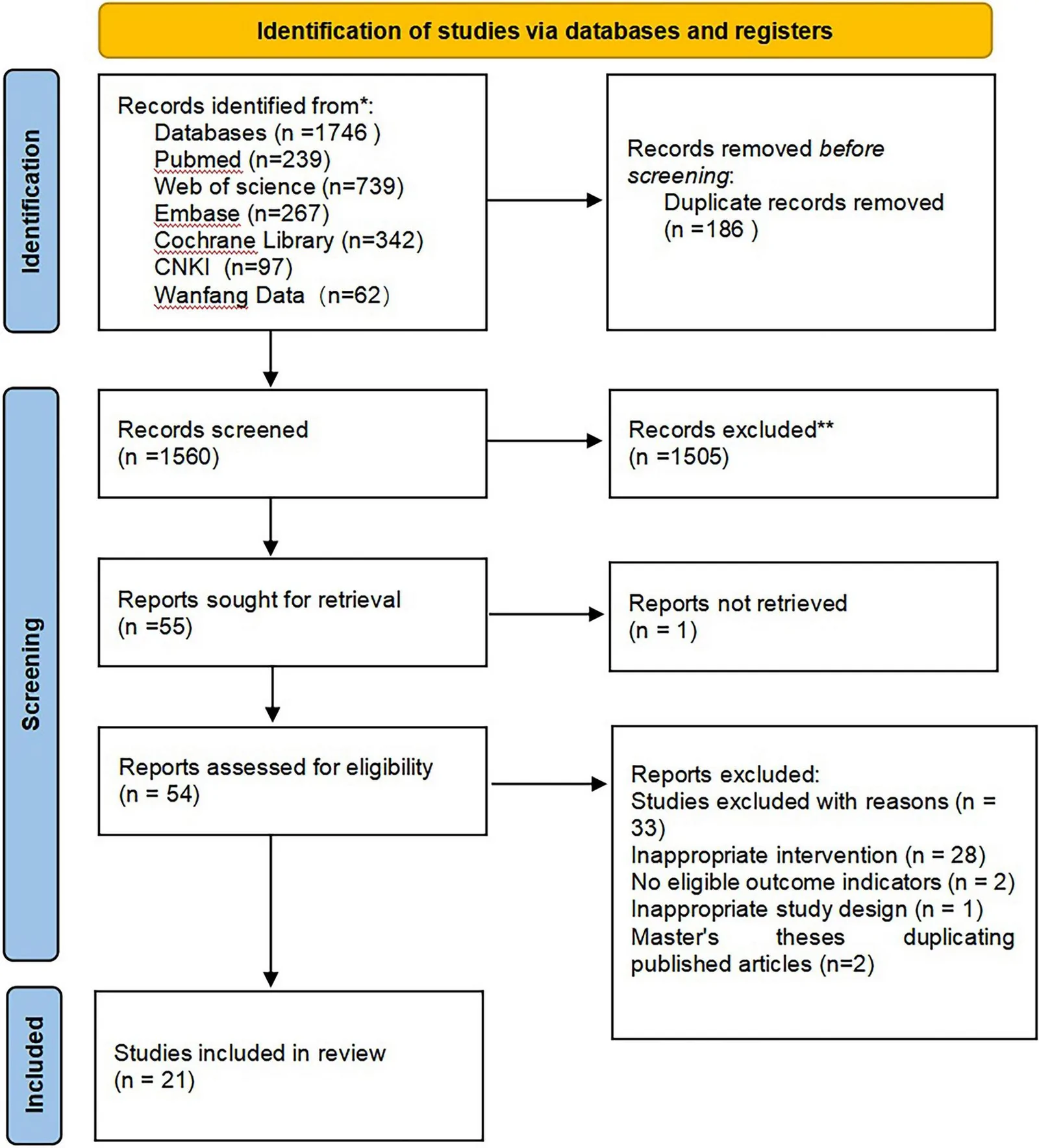
PRISMA flow diagram.

A total of 1,746 records were retrieved from six databases: PubMed (239), Web of Science (739), Embase (267), Cochrane Library (342), CNKI (97), and Wanfang Medical Network (62). After removing 186 duplicates, 1,560 unique records underwent title and abstract screening, and 1,505 irrelevant records were excluded. Full texts were sought for the remaining 55 articles, while one publication was unobtainable despite multiple retrieval attempts. The remaining 54 full-text articles were assessed for eligibility, and 33 were excluded for predefined reasons: 28 with ineligible interventions, 2 without target outcomes, 1 non-RCT, and 2 master’s theses. Ultimately, 21 RCTs (Cao et al., 2016; Chen, 2022; Fan and Yi, 2018; Fan et al., 2025; Jin et al., 2024; Liu et al., 2020; Liu et al., 2025; Lu and Pang, 2019; Lu, 2024; Ma et al., 2025; Niu et al., 2015; Pu et al., 2021; Tian and Xue, 2024; Wang et al., 2020; Wang et al., 2022; Wang, 2022; Xi et al., 2023; Zhang et al., 2021; Zhang et al., 2025; Zhao et al., 2025; Zhu et al., 2017) with a total of 1748 stroke patients were included. The screening procedure is summarized in the PRISMA flow diagram (Figure 1).

### 3.2 Characteristics of included studies

Twenty-one RCTs with a total of 1,748 stroke adults were included, mostly enrolling ischemic stroke survivors aged 50–70 years with more male participants. Trials covered acute (≤ 14 days) and non-acute (> 14 days) stroke phases, with wide variation in baseline motor, cognitive and daily living impairment severity. All participants received conventional rehabilitation as basic treatment; the experimental group received supplementary TUS combined intervention, while the control group received routine rehabilitation or sham stimulation.

Substantial between-study heterogeneity was observed in TUS parameters: 800 kHz frequency and 0.75 W/cm^2^ intensity were most widely used, with single sessions lasting 15–30 min and multi-target temporal window stimulation as the mainstream protocol. Many critical pulse-related parameters were incompletely reported. Treatment cycles ranged from 10 days to 6 weeks, and primary outcomes included neurological, motor, cognitive, daily living and swallowing function scales.

The basic characteristics of the included studies are presented in Table 1, and the TUS parameters are detailed in Table 2.

**TABLE 1:** Baseline Characteristics of Included Studies.

Author	Year	Disease duration	Sample size	Age (years, mean ± SD)	Sex (M/F, n)	Treatment course	Intervention		Reported outcomes
							Experimental group	Control group	
Zhao et al. (2025)	2025	≥ 2 weeks	90	62. 1 ± 10. 2	52/38	4weeks	TUS, PT, OT	PT, OT	MMSE, SFMA, MBI
Wang (2022)	2022	<6 h	90	(61.32 ± 2.14)	50/40	3 weeks	TCM, PTa, TUS	TCM	FMA, ADL, MESSS
Zhang et al. (2021)	2021	>1 month	60	67.20 ± 17.38	38/22	6 weeks	OT, TUS	OT+ sham TUS	MMSE, MBI
Fan and Yi (2018)	2018	–	80	–	–	20 days	E + TUS	E	Fujishima-Ichiro Dysphagia
Zhu et al. (2017)	2017	–	82	–	45/37	4 weeks	PTb, Med, TUS	Med	Barthel, Fujishima-Ichiro Dysphagia
Fan et al. (2025)	2025	>10 days	103	69.52 ± 6.25	43/60	20 days	Med, TUS	Med	NIHSS, MMSE,
Lu (2024)	2024	>20 days	60	61.6 ± 4.2	34/26	3 weeks	Med, PTa, TCM, TUS	Med, PTa, TCM	FMA, MoCA,
Ma et al. (2025)	2025	1–3 months	46	60.30 ± 10.33	23/23	4 weeks	PTa, PTb, E, TUS	PTa, PTb, E	FMA-LE, BBS, FAC, MBI
Pu et al. (2021)	2021	<3 months	114	55.55 ± 4.40	39/37	4 weeks	Med, PTa, E, TUS	Med, PTa, E	FMA, MBI
Tian and Xue (2024)	2024	–	52	59.92 ± 3.32	29/23	2 months	E, TUS	E	VFSS, SSA, NIHSS
Wang et al. (2020)	2020	<3 months	45	54.78 ± 6.83	15/15	4 weeks	Med, PTa, OT, E, TUS	Med, PTa, OT, E	FMA, MBI,
Xi et al. (2023)	2023	> half month	30	50.9 ± 15.1	7月23日	2 weeks	Med, PTa, OT, TUS	Med, PTa, OT, sham TUS	FMA-UE, WMFT, JHFT, BI, NIHSS
Zhang et al. (2025)	2025	–	68	69.28 ± 6.23	38/30	4 weeks	Med, E, TUS	Med, E	FMA-UE, MSS, BBS
Jin et al. (2024)	2024	<3 months	109	59.93 ± 6.79	60/49	3 weeks	Med, PTa, OT, E, TUS	Med, PTa, OT, E	FMA, MBI, FAC, Brunnstrom
Liu et al. (2020)	2020	–	60	64.50 ± 2.53	37/23	4 weeks	PTb, TUS	PTb	NIHSS, FMA
Lu and Pang (2019)	2019	–	90	63.5 ± 4.9	49/41	4 weeks	Med, PTa, TUS	Med, PTa	VAS, FMA, BI, ADL
Niu et al. (2015)	2015	<6 h	137	69.2 ± 3.3	78/59	4 weeks	Med, PTa, TUS	Med, PTa	FMA, MBI
Chen (2022)	2022	>48 h	60	59.4 ± 3.3	37/23	4 weeks	RNC, TUS	RNC	BI, ESS
Cao et al. (2016)	2016	–	62	62.1 ± 11.2	36/26	1.5 weeks	Med, TCM, RNC, PTa, TUS	Med, TCM, RNC, PTa	FMA, BI
Wang et al. (2022)	2022	>1 month	60	57.73 ± 7.90	Dec-48	6 weeks	OT + TUS	OT + sham TUS	, MoCA, MBI, P300
Liu et al. (2025)	2025	<2 weeks	90	56. 00 ± 9. 00	36/24	1-2 weeks	Med, PTa, PTb, TUS	Med, PTa, PTb	sFMA, BI

PTa, motor therapy; PTb, physical modality therapy; OT, occupational therapy; TCM, traditional Chinese medicine therapy; RNC, routine nursing care; Med, conventional medication; E, other rehabilitation therapies. ADL, activities of daily living; BBS, Berg Balance Scale; BI, Barthel Index; ESS, European Stroke Scale; FAC, Functional Ambulation Category; FMA, Fugl-Meyer Assessment (FMA-UE for upper extremity, FMA-LE for lower extremity); JHFT, Jebsen–Taylor Hand Function Test; MBI, Modified Barthel Index; MESSS, Modified Edinburgh–Scandinavian Stroke Scale; MMSE, Mini-Mental State Examination; MoCA, Montreal Cognitive Assessment; MSS, Motor Status Scale; NIHSS, National Institutes of Health Stroke Scale; P300, P300 event-related potential; SSA, Standardized Swallowing Assessment; SFMA, Selective Functional Movement Assessment; VAS, Visual Analog Scale; VFSS, Videofluoroscopic Swallowing Study; WMFT, Wolf Motor Function Test.

**TABLE 2:** TUS Parameters in included studies.

Author	Area (cm^2^)	Frequency (kHz)	Intensity (W/cm^2^)	Pulse duration (ms)	Repetition period (ms)	Duty cycle (%)	Session duration (min)	Placement location
Zhao et al. (2025)	3	700	0.65	–	–	–	20	A
Wang (2022)	–	–	–	–	–	–	30	A, C
Zhang et al. (2021)	2	800	1.75	–	–	–	20	–
Fan and Yi (2018)	–	–	–	–	–	–	20	A, B, C
Zhu et al. (2017)	–	800	0.6∼1.25	–	–	1:3	30	A, C
Fan et al. (2025)	–	800	1.2	17.5	25	–	30	A, C
Lu (2024)	–	800	0.6∼1.25	–	–	–	30	A, B, C
Ma et al. (2025)	–	800	0.54	–	–	–	20	A, B
Pu et al. (2021)	≤ 2	800	1.2	0∼100 μs	25	10∼100	20	A, B
Tian and Xue (2024)	–	–	–	–	–	–	20	A, B
Wang et al. (2020)	< 2	800	1.2	17.5	25	10∼100	20	A, B, F
Xi et al. (2023)	–	–	0.75	–	–	50	15	E
Zhang et al. (2025)	–	600	1.5	–	25	50∼80	1 0∼15	A, B
Jin et al. (2024)	–	–	–	–	–	–	30	A, B
Liu et al. (2020)	3	800∼880	0.75	10	60∼150	–	15	A, B
Lu and Pang (2019)	3	800 ± 80	0.75	10	60∼150	100%	15	A, B
Niu et al. (2015)	3	800 ± 80	0.75	–	–	100%	30	A
Chen (2022)	9	800	0.75	–	–	–	20	A
Cao et al. (2016)	3	800	0.75	–	–	–	20	G
Wang et al. (2022)	2	–	1.75	–	–	–	20	D
Liu et al. (2025)	3∼4	800	0.9	10	60∼150	1:5∼1:14	20	A, B, H

A, ipsilesional temporal window; B, contralesional temporal window; C, surface projection area of vertebral artery, internal carotid artery, and lesion; D, forehead; E, target treatment area; F, brain injury site; G, site determined based on occluded vessel; H, occipital window.

### 3.3 Quality of included studies

**FIGURE 2:**
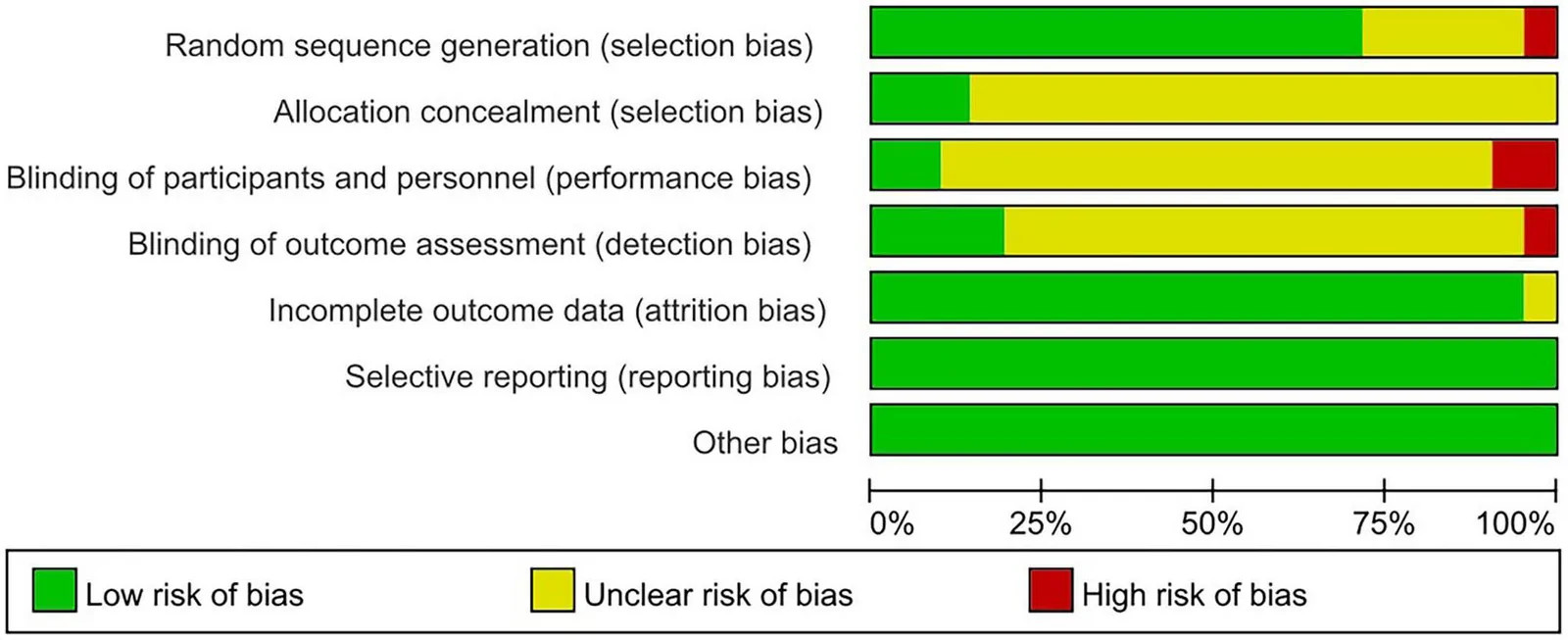
Risk of bias graph.

**FIGURE 3:**
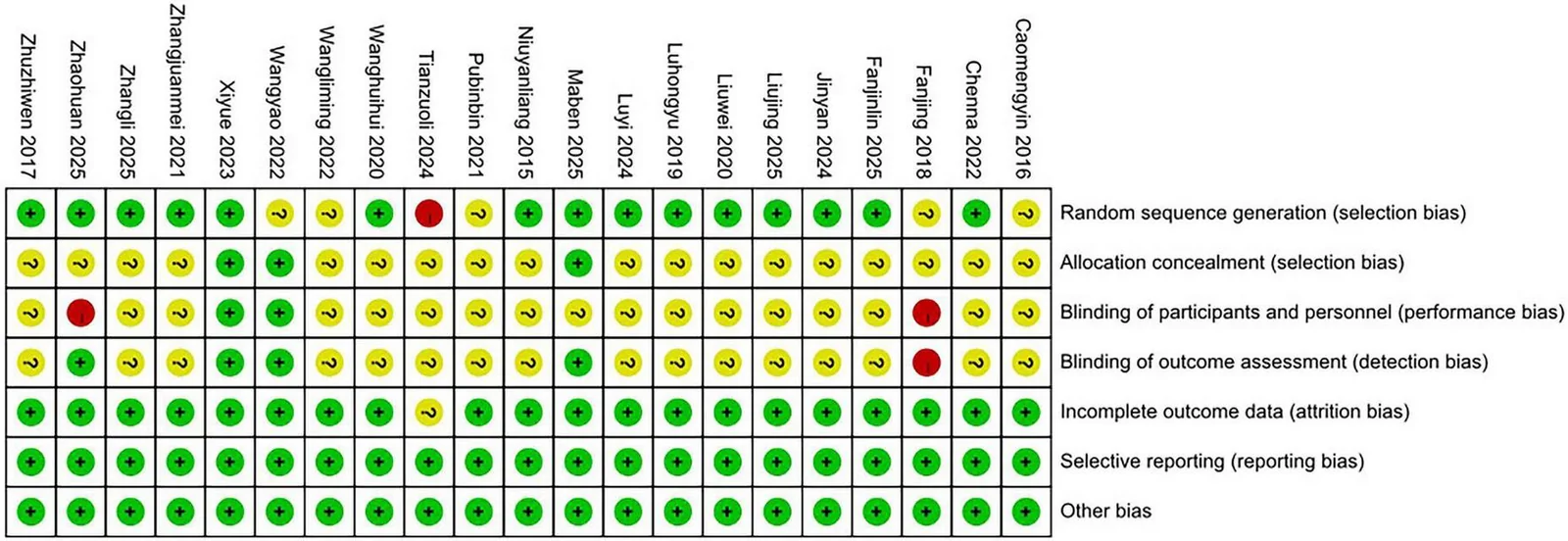
Risk of bias summary.

The Cochrane Risk of Bias assessment showed that most trials had low risk of bias for random sequence generation and incomplete outcome data, but allocation concealment and blinding of participants and personnel were often inadequately reported or judged as high risk, resulting in an overall moderate risk of bias across studies. The risk of bias graph is shown in Figure 2, and the risk of bias summary is shown in Figure 3.

Methodological quality of the 21 included randomized controlled trials (RCTs) was evaluated using the PEDro scale, and the detailed scoring results are presented in Table 3. The total PEDro scores of all studies ranged from 4 to 7. High-quality studies (score ≥ 7): 3 articles. Moderate-quality studies (score 4–6): 18 articles, including 15 trials with 5 points, 3 trials with 6 points and 1 trial with 4 points.

**TABLE 3:** Methodological quality assessment of included RCTs using the PEDro scale.

Study	Eligibility criterion	Random allocation	Concealed allocation	Baseline similarity	Blinding of subjects	Blinding of therapists	Blinding of assessors	Follow-up > 85%	Intention to treat	Between-group compa- risons	Point and variability measures	Total (0—-10)	Scores
Zhao et al. (2025)	Y	Y	N	Y	N	N	Y	Y	N	Y	Y	6	Good
Wang (2022)	Y	Y	N	Y	N	N	N	Y	N	Y	Y	5	Fair
Zhang et al. (2021)	Y	Y	N	Y	Y	N	N	Y	N	Y	Y	6	Good
Fan and Yi (2018)	N	Y	N	Y	N	N	N	Y	N	Y	Y	5	Fair
Zhu et al. (2017)	Y	Y	N	Y	N	N	N	Y	N	Y	Y	5	Fair
Fan et al. (2025)	Y	Y	N	Y	N	N	N	Y	N	Y	Y	5	Fair
Lu (2024)	Y	Y	N	Y	N	N	N	Y	N	Y	Y	5	Fair
Ma et al. (2025)	Y	Y	N	Y	Y	N	Y	Y	N	Y	Y	7	Good
Pu et al. (2021)	Y	Y	N	Y	N	N	N	Y	N	Y	Y	5	Fair
Tian and Xue (2024)	Y	N	N	Y	N	N	N	Y	N	Y	Y	4	Fair
Wang et al. (2020)	Y	Y	N	Y	N	N	N	Y	N	Y	Y	5	Fair
Xi et al. (2023)	Y	Y	N	Y	Y	N	Y	Y	N	Y	Y	7	Good
Zhang et al. (2025)	Y	Y	N	Y	N	N	N	Y	N	Y	Y	5	Fair
Jin et al. (2024)	Y	Y	N	Y	N	N	N	Y	N	Y	Y	5	Fair
Liu et al. (2020)	Y	Y	N	Y	N	N	N	Y	N	Y	Y	5	Fair
Lu and Pang (2019)	Y	Y	N	Y	N	N	N	Y	N	Y	Y	5	Fair
Niu et al. (2015)	Y	Y	N	Y	N	N	N	Y	N	Y	Y	5	Fair
Chen (2022)	Y	Y	N	Y	N	N	N	Y	N	Y	Y	5	Fair
Cao et al. (2016)	Y	Y	N	Y	N	N	N	Y	N	Y	Y	5	Fair
Wang et al. (2022)	Y	Y	N	Y	Y	N	Y	Y	N	Y	Y	7	Good
Liu et al. (2025)	Y	Y	N	Y	N	N	N	Y	N	Y	Y	5	Fair

Y, Yes; N, No.

### 3.4 Results of meta-analysis

Predefined subgroup analyses were conducted with standardized grouping thresholds formulated prior to data extraction. Stroke phase was stratified as acute (onset ≤ 14 days) and non-acute (onset > 14 days) in accordance with mainstream post-stroke rehabilitation standards. Baseline Barthel Index scores were used to classify functional dependence: moderate dependence (41–60 points) and severe dependence (≤ 40 points). All participants from included trials were reclassified uniformly based on the above unified criteria to eliminate inconsistent definition standards adopted across individual original studies, and inter-subgroup difference tests were performed to compare pooled effect estimates.

#### 3.4.1 NIHSS score

**FIGURE 4:**
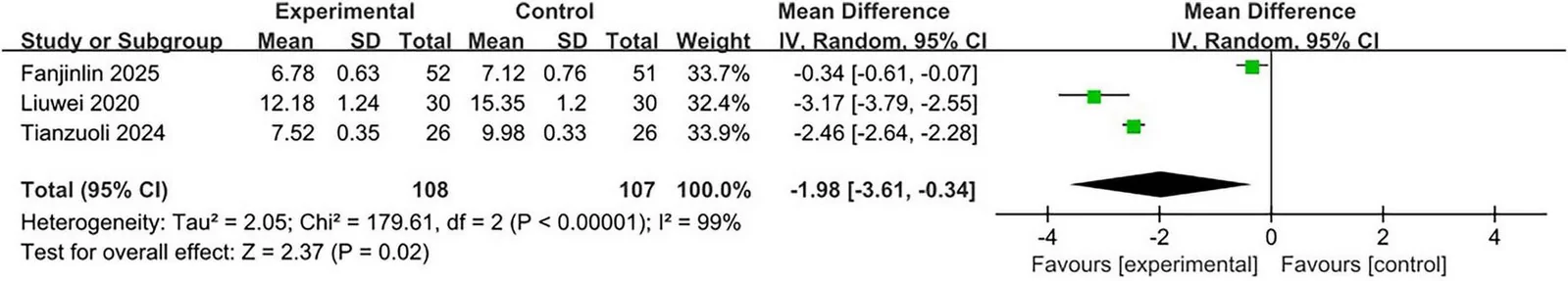
Forest plot of NIHSS score: TUS combination vs. control.

Three RCTs (Fan et al., 2025; Liu et al., 2025; Tian and Xue, 2024) involving 215 patients were included. The pooled result using a random-effects model showed that the TUS combination group had a significantly lower NIHSS score compared to the control group (MD = −1.98, 95% CI: −3.61 to −0.34, *P* = 0.02), with high heterogeneity among studies (*I*^2^ = 99%, *P* < 0.001). This high heterogeneity might be due to differences in study populations, interventions, and stroke severity/disease phase across the three studies. Despite this, all three studies reported significantly greater reductions in NIHSS scores in the intervention group compared to the control group (all *P* < 0.05). It is important to note that, due to substantial variation in baseline neurological deficit severity (NIHSS scores ranging from approximately 9 to 20 points), the between-group differences in improvement also varied, ranging from −0.34 to −3.17 points. Results are shown in Figure 4.

#### 3.4.2 MMSE score

Four RCTs (Fan et al., 2025; Wang et al., 2022; Zhang et al., 2021; Zhao et al., 2025) involving 313 patients were included. Using a random-effects model, the TUS combination group showed significantly higher MMSE scores than the control group (MD = 1.22, 95% CI: 0.74–1.71, *P* < 0.001).

**FIGURE 5:**
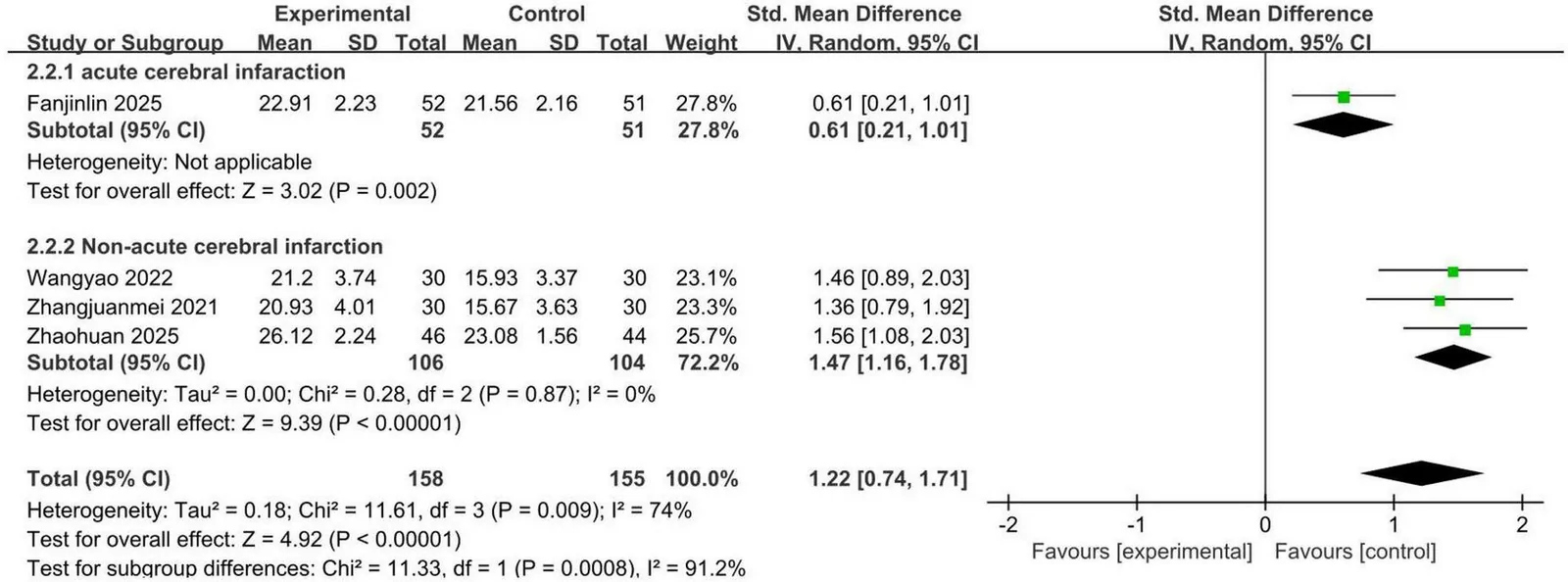
Forest plot of MMSE score: TUS combination vs. control.

Subgroup analysis for the MMSE score was performed due to high overall heterogeneity (*I*^2^ = 74%). Studies were divided into non-acute phase cerebral infarction and acute cerebral infarction subgroups. Heterogeneity decreased in the non-acute phase subgroup (*I*^2^ = 0%), with a pooled effect size of (MD = 1.47, 95% CI: 1.16–1.78, *P* < 0.001). The acute-phase subgroup included only one study (MD = 0.61, 95% CI: 0.21–1.01). The difference between subgroups was statistically significant (*P* < 0.001). Results are shown in Figure 5.

#### 3.4.3 FMA scale score

**FIGURE 6:**
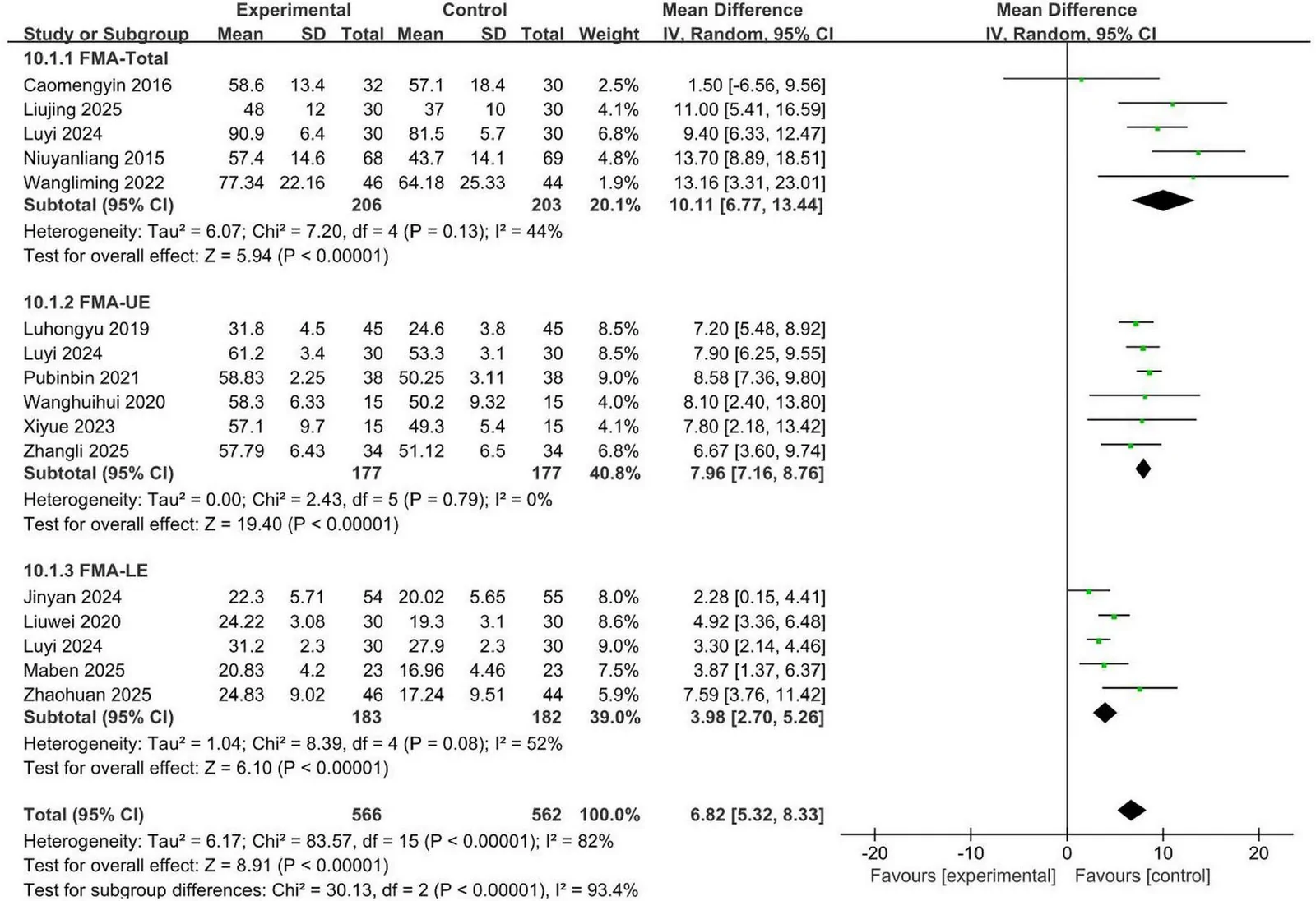
Forest plot of FMA score: TUS combination vs. control.

Fourteen RCTs (Cao et al., 2016; Liu et al., 2025; Lu and Pang, 2019; Lu, 2024; Pu et al., 2021; Wang et al., 2020; Wang, 2022; Xi et al., 2023; Zhang et al., 2025) involving 1,128 patients were included. Using a random-effects model, subgroup analysis showed that the intervention markedly improved Fugl-Meyer motor function scores, with effects varying by assessment site. For upper limb function, the TUS combination group had significantly higher FMA-UE scores than the control group (MD = 7.96, 95% CI: 7.16–8.76, *P* < 0.001). For lower limb function, the experimental group had substantially higher FMA-LE scores than the control group (MD = 3.98, 95% CI: 2.70–5.26, *P* < 0.001). For total motor function, the experimental group had significantly higher total FMA scores than the control group (MD = 10.11, 95% CI: 6.77–13.44, *P* < 0.001). The difference between subgroups was statistically significant (*P* < 0.001). Results are shown in Figure 6.

#### 3.4.4 Barthel Index (BI) score

Eleven RCTs (Cao et al., 2016; Chen, 2022; Jin et al., 2024; Liu et al., 2025; Lu and Pang, 2019; Niu et al., 2015; Pu et al., 2021; Wang et al., 2020; Wang et al., 2022; Zhang et al., 2021; Zhao et al., 2025) involving 834 patients were included. Using a random-effects model, the TUS combination group had significantly higher BI scores than the control group (MD = 9.08, 95% CI: 5.72–12.44, *P* < 0.001), with high heterogeneity among studies (*I*^2^ = 89%, *P* < 0.001). Considering that baseline BI scores and different interventions might be major sources of heterogeneity, subgroup analyses were performed.

**FIGURE 7:**
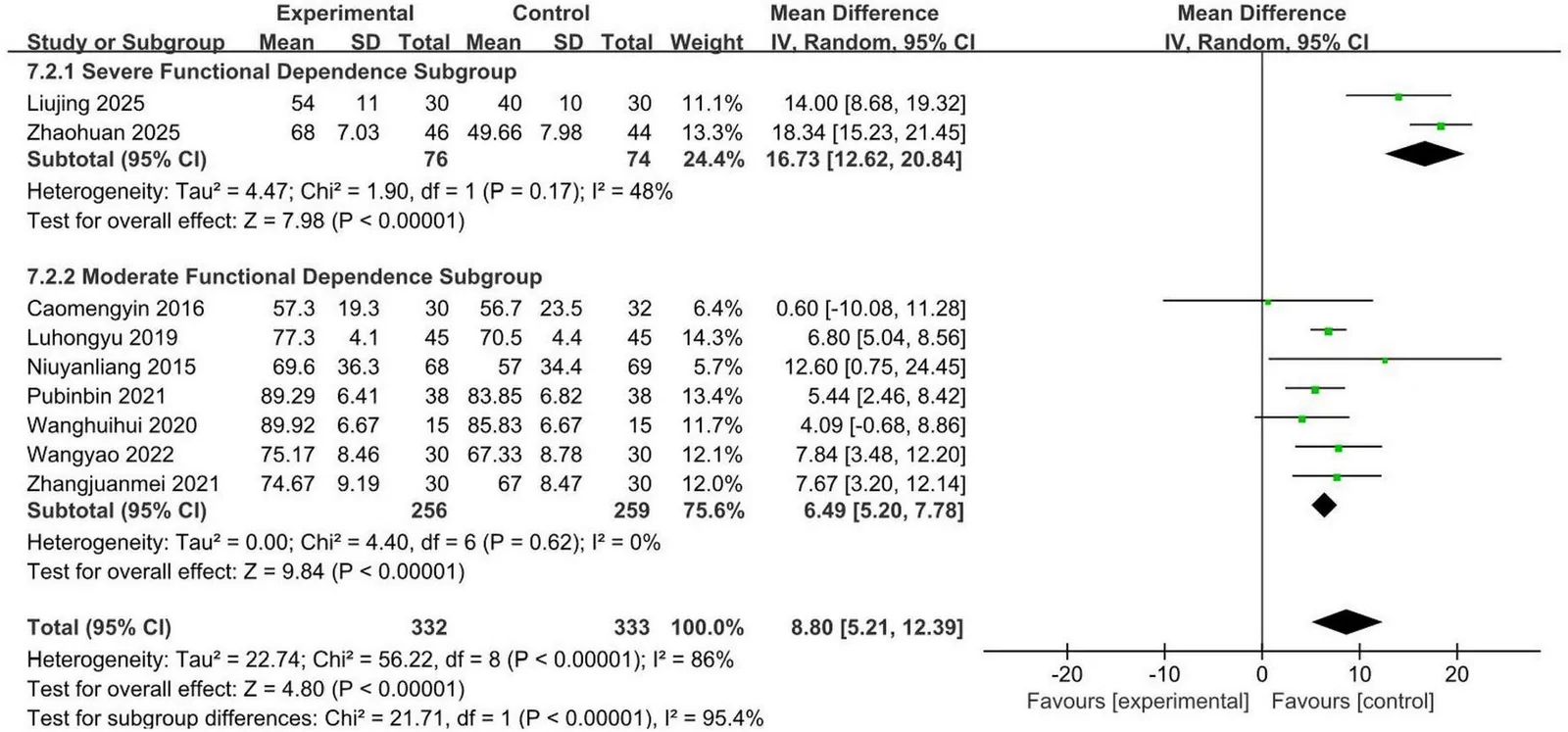
Forest plot of BI score: TUS combination vs. control.

Subgroup analysis divided patients into moderately dependent and severely dependent subgroups. Heterogeneity was high in the moderately dependent subgroup (*I*^2^ = 81%). Sensitivity analysis revealed that excluding Chen Na (Liu et al., 2020) (whose control group received only detailed nursing care, differing from conventional rehabilitation in other studies) reduced heterogeneity significantly in this subgroup (*I*^2^ from 81 to 0%), showing significantly higher scores in the experimental group (MD = 6.94, 95% CI: 5.20–7.78, *P* < 0.01). Heterogeneity remained high in the severely dependent subgroup (*I*^2^ = 97%). Sensitivity analysis showed that excluding Jin Yan (Wang et al., 2020) (whose control group received Xingnao Kaiqiao acupuncture, differing from conventional basic treatment in other studies) reduced heterogeneity significantly in this subgroup (*I*^2^ from 94 to 48%), showing significantly higher scores in the experimental group (MD = 16.73, 95% CI: 12.62–20.84, *P* < 0.01). Results are shown in Figure 7.

#### 3.4.5 Fujishima-Ichiro dysphagia score

**FIGURE 8:**
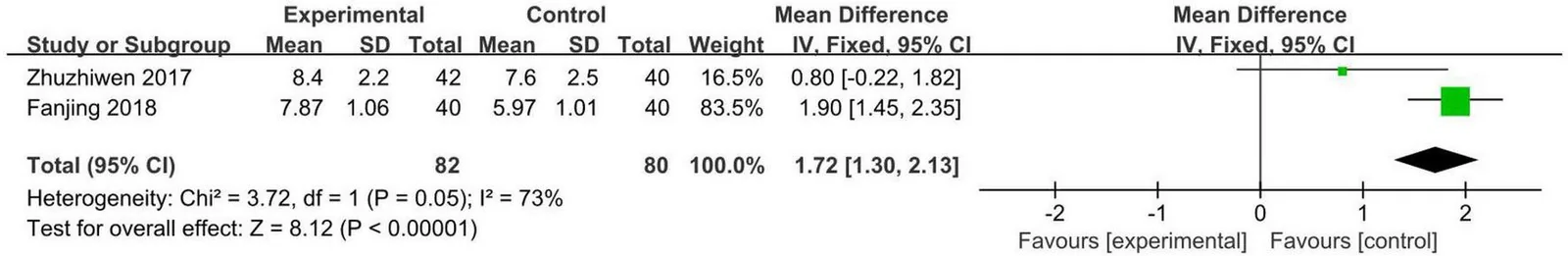
Forest plot of Fujishima-Ichiro score: TUS combination vs. Control.

Two RCTs (Fan and Yi, 2018; Zhu et al., 2017) involving 162 patients were included. Using a random-effects model, the TUS combination group had significantly higher Fujishima-Ichiro Dysphagia Scores than the control group (MD = 1.72, 95% CI: 1.30–2.13, *P* < 0.001). Results are shown in Figure 8.

#### 3.4.6 Subgroup analyses of TUS parameters

To explore potential sources of heterogeneity and to evaluate whether TUS treatment effects vary by technical parameters, we performed additional subgroup analyses stratified by ultrasound frequency, intensity, stimulation site and session duration for both the Barthel Index (BI) and Fugl-Meyer Assessment (FMA) outcomes. It should be noted that subgroup analyses for TUS session duration in the BI outcome, as well as for session duration, intensity, and stimulation site in the FMA outcome, were precluded by persistently high statistical heterogeneity (*I*^2^ > 75%) that could not be adequately resolved through sensitivity analyses or clinical re-stratification; these analyses were therefore considered unsuitable for quantitative synthesis and are not reported in this section.

Sensitivity analysis showed that excluding (Liu et al., 2025) and (Ma et al., 2025) markedly reduced heterogeneity in the corresponding TUS parameter subgroup analyses. The heterogeneity was most likely driven by these two trials, which incorporated fastigial nucleus electrical stimulation or lower-limb robot training as combined interventions, differing from other studies that only adopted TUS monotherapy alongside routine rehabilitation.

##### 3.4.6.1 Subgroup analysis of BI score by TUS parameters

Frequency subgroup analysis: 7 studies were categorized into low-frequency (< 800 kHz) and 800 kHz groups. The low-frequency group included 1 study with an MD of 18.34 (95% CI: 15.23–21.45, *P* < 0.001, *I*^2^ = 0%), while the 800 kHz group included 6 studies with a pooled MD of 6.36 (95% CI: 5.00–7.71, *P* < 0.001, *I*^2^ = 0%). The between-group difference was statistically significant (*P* < 0.001).

Intensity subgroup analysis: 8 studies were divided into < 0.75 W/cm^2^, 0.75 W/cm^2^, and > 0.75 W/cm^2^ groups. The < 0.75 W/cm^2^ group included 1 study with an MD of 18.34 (95% CI: 15.23–21.45). The 0.75 W/cm^2^ group included 3 studies with a pooled MD of 6.70 (95% CI: 3.74–9.65, *P* < 0.001, *I*^2^ = 10%). The > 0.75 W/cm^2^ group included 4 studies with an MD of 6.13 (95% CI: 4.16–8.09, *P* < 0.001, *I*^2^ = 0%). The between-group difference was statistically significant (*P* < 0.001).

**FIGURE 9:**
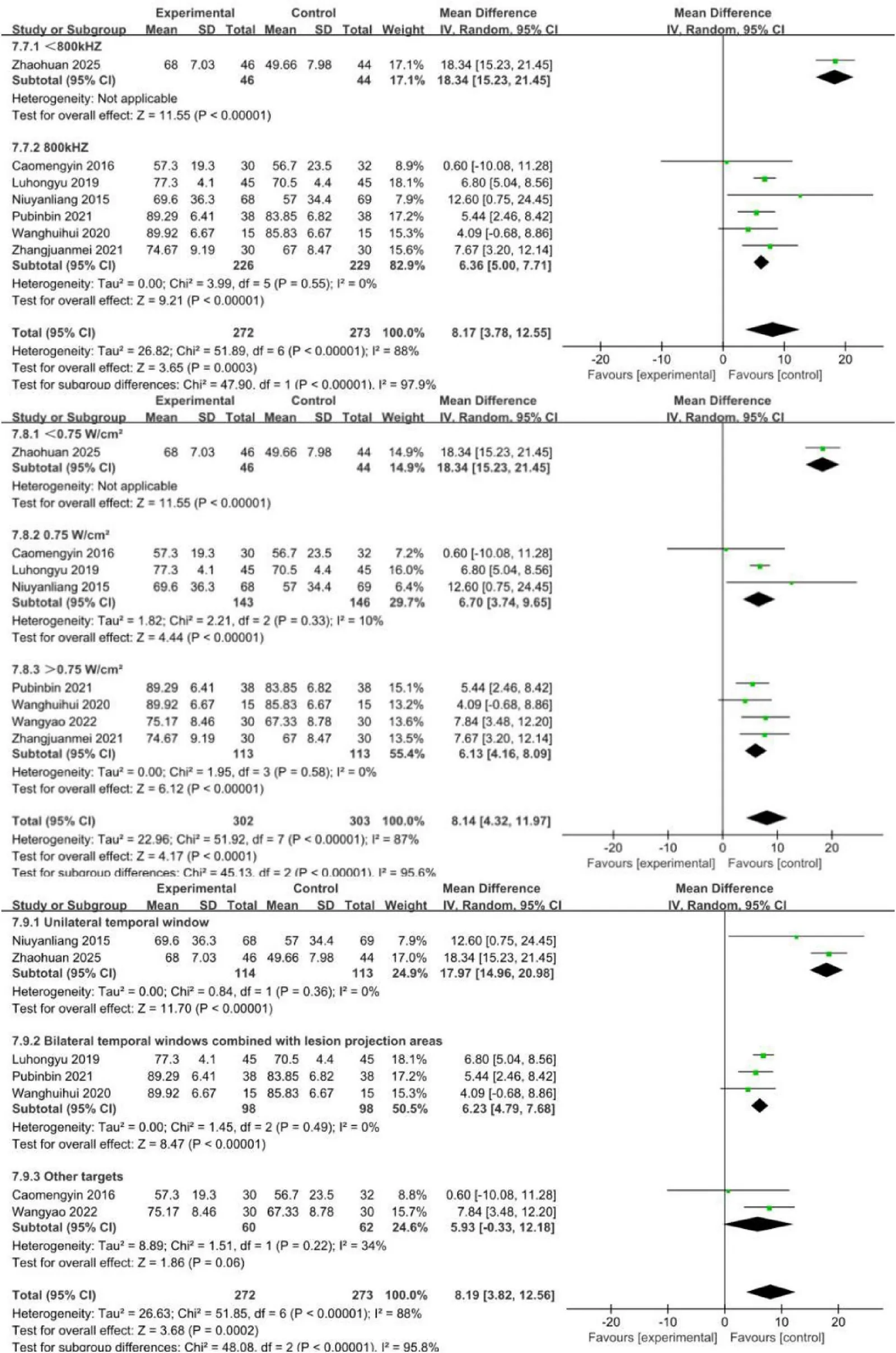
Forest plots of BI score subgroup analyses by TUS frequency, intensity, and stimulation site.

Stimulation site subgroup analysis: 7 studies were grouped into unilateral temporal window, bilateral temporal windows combined with lesion projection areas, and other stimulation targets. The unilateral temporal window group included 2 studies with a pooled MD of 17.97 (95% CI: 14.96–20.98, *P* < 0.001, *I*^2^ = 0%), The bilateral temporal windows combined with lesion projection areas group included 3 studies with a pooled MD of 6.23 (95% CI: 4.79–7.68, *P* < 0.001, *I*^2^ = 0%). The other stimulation targets group included 2 studies with a pooled MD of 5.93 (95% CI: −0.33 to 12.18, *P* = 0.06, *I*^2^ = 34%), which failed to reach statistical significance. The between-group difference was statistically significant (*P* < 0.001). Results are presented in Figure 9.

##### 3.4.6.2 Subgroup analysis of FMA score by TUS frequency

**FIGURE 10:**
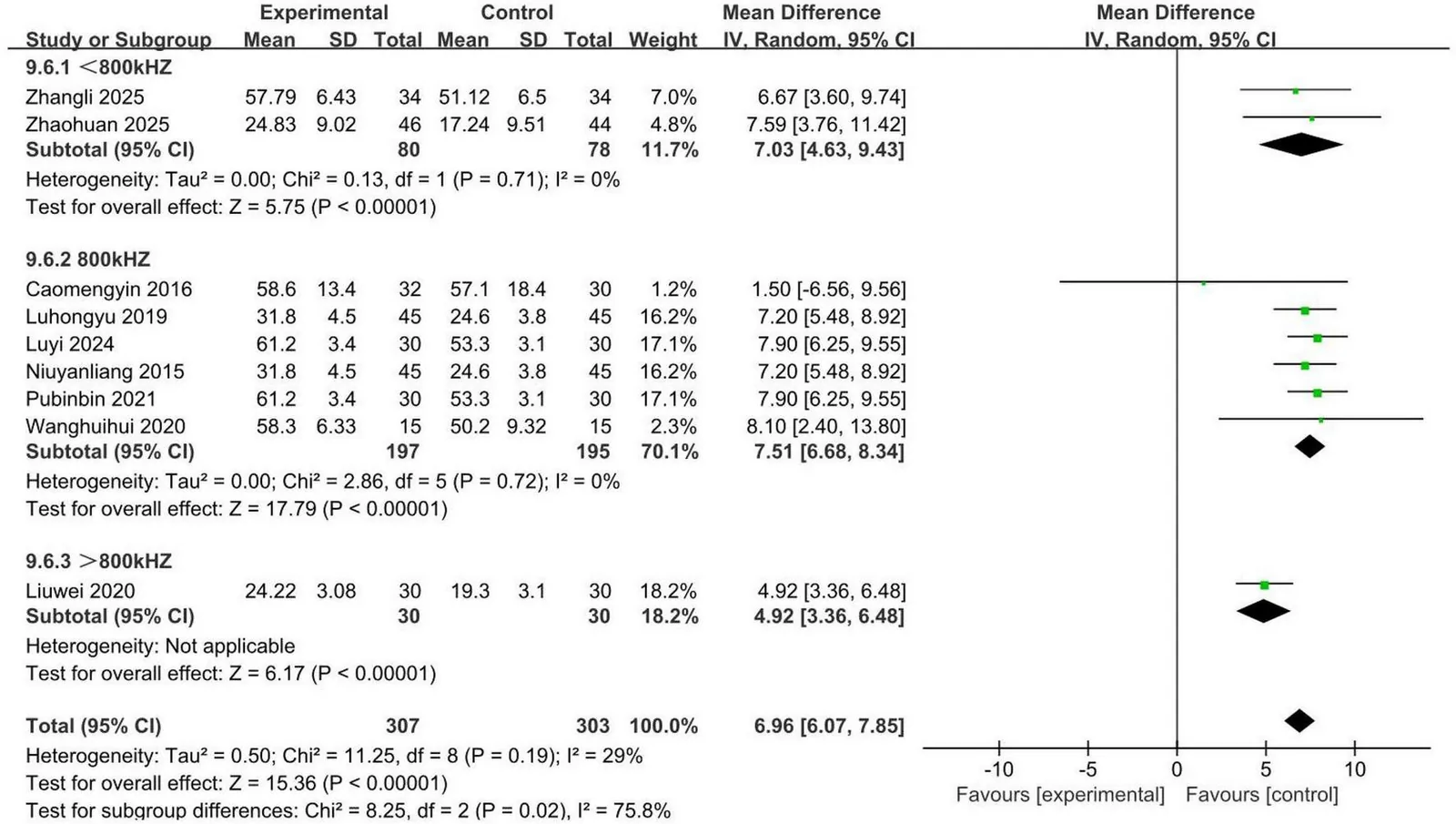
Forest plots of FMA score subgroup analyses by TUS frequency.

Nine studies were categorized into low-frequency (<800 kHz), 800 and > 800 kHz groups. The < 800 kHz group included 2 studies with a pooled MD of 7.03 (95% CI: 4.63–9.43, *P* < 0.001, *I*^2^ = 0%). The 800 kHz group included 6 studies with a pooled MD of 7.51 (95% CI: 6.68–8.34, *P* < 0.001, *I*^2^ = 0%). The > 800 kHz group included 1 study with an MD of 4.92 (95% CI: 3.36–6.48, *P* < 0.001). The between-group difference was statistically significant. Results are presented in Figure 10.

### 3.5 Risk of bias in included studies

**FIGURE 11:**
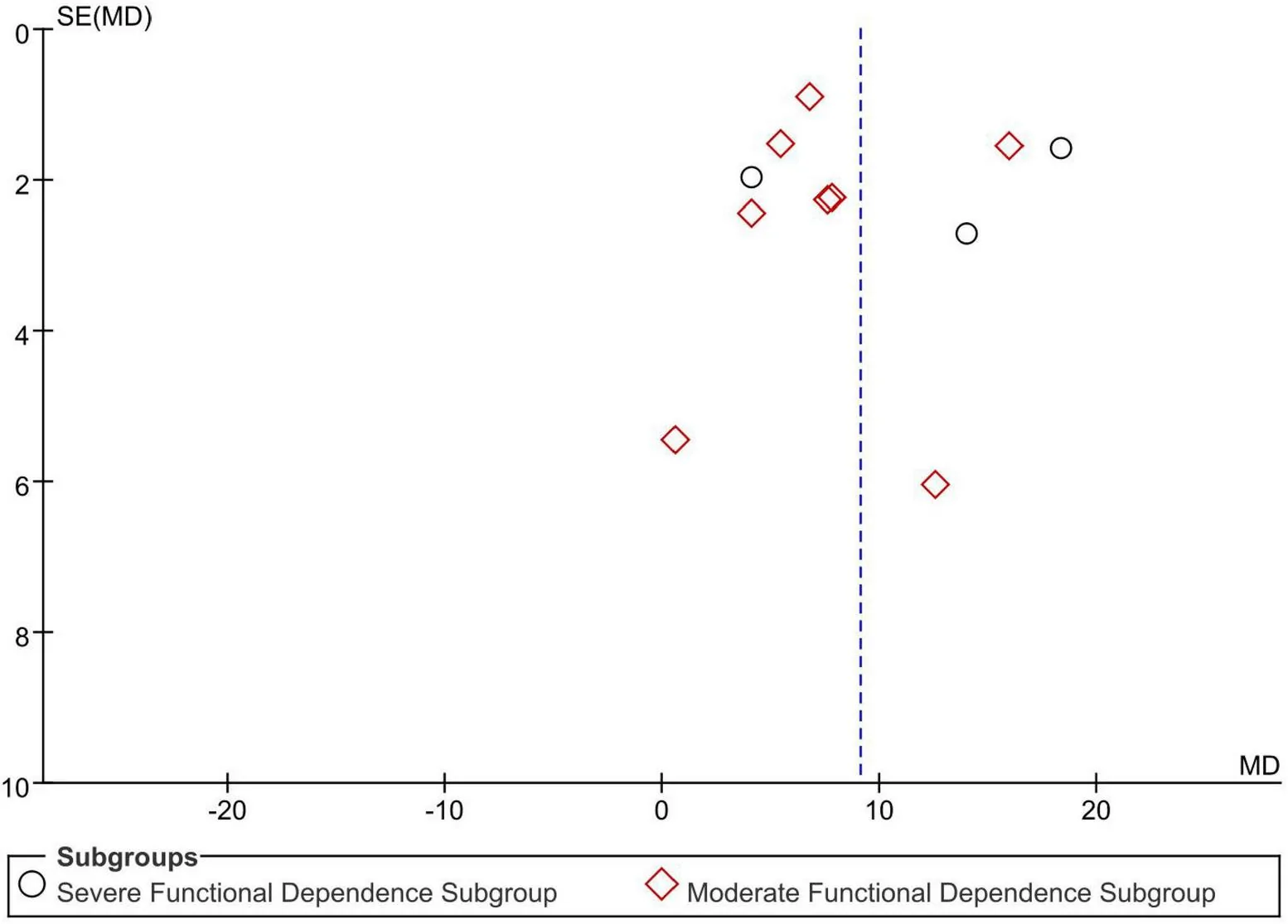
Funnel plot assessing publication bias for the barthel index (BI) score.

A funnel plot was generated, and Egger’s test was performed for the outcome with the most included studies (BI score, 11 studies). The funnel plot showed roughly symmetrical distribution of study points (Figure 11), and Egger’s test indicated no statistically significant publication bias (*P* = 0.62 > 0.05).

## 4 Discussion

By integrating clinical evidence from 21 randomized controlled trials involving 1,748 patients, this meta-analysis confirmed that transcranial ultrasound stimulation (TUS) adjunctive to conventional rehabilitation significantly improves neurological deficits, upper and lower limb motor function, cognitive function, swallowing function, and activities of daily living in stroke survivors. This finding supports the clinical value of TUS as an auxiliary rehabilitation intervention from an evidence-based perspective.

### 4.1 Mechanisms and clinical translation

The neuromodulatory effects of transcranial ultrasound stimulation (TUS) operate through various pathways and targets. Preclinical studies have shown that TUS dilates cerebral blood vessels and enhances regional cerebral perfusion through mechanical and thermal effects. Additionally, it regulates ion channels and neurotransmitter release, suppresses neuroinflammation, and improves synaptic plasticity, which helps reorganize damaged neural networks (Chen et al., 2018; Guo et al., 2022; Ichijo et al., 2021; Saguchi et al., 2008; Shen et al., 2018; Wang et al., 2021). Clinical findings support the relevance of these mechanistic observations. For example, patients in the TUS combined group demonstrated significantly lower NIHSS scores compared to the control group. Although the absolute difference between groups may seem modest, a reduction of 2 points in the NIHSS is clinically significant for patients in the acute phase. It could indicate a functional shift from severe to moderate impairment or from moderate to mild impairment, thus assisting in the timing and selection of early rehabilitation strategies.

In terms of motor recovery, transcranial ultrasound (TUS) resulted in greater improvements in upper-extremity Fugl-Meyer scores (FMA-UE) compared to lower-extremity outcomes. This difference is attributed to the distinct cortical representation and inherent plasticity of the motor areas for the upper and lower limbs (Sobinov and Bensmaia, 2021; Zeng et al., 2021; Zhang et al., 2022). Understanding this disparity provides valuable guidance for designing clinical TUS protocols. TUS is preferred for patients with a predominant fine hand motor deficit. For individuals with primary gait and lower-limb dysfunction, TUS should be combined with supplementary interventions such as lower-limb robotic training or balance exercises (Ma et al., 2025). Clinically, physical or occupational therapy should be administered immediately following TUS, leveraging the transient cortical excitability window induced by ultrasound to amplify the gains of subsequent rehabilitation training.

### 4.2 Heterogeneity and effect attribution

Substantial statistical heterogeneity was observed across multiple outcome indicators in this meta-analysis. In-depth subgroup and sensitivity analyses identified two major contributing factors: discrepancies in patient clinical profiles (e.g., stroke phase, baseline functional severity) and variable composite intervention protocols. Although all experimental arms across the 21 included trials used TUS plus conventional rehabilitation, the composition of baseline rehabilitation varied widely, encompassing combinations of kinesiotherapy, physical agents, occupational therapy, acupuncture, and routine nursing care. Several studies (Chen, 2022; Jin et al., 2024; Liu et al., 2025) further superimposed additional active interventions, including Xingnao Kaiqiao acupuncture and fastigial nucleus electrical stimulation alongside standard rehabilitation. Consequently, the pooled effect sizes reported herein reflect the composite therapeutic benefit of TUS added onto heterogeneous rehabilitation frameworks, rather than the standalone net efficacy of TUS monotherapy.

This interpretation bears dual clinical and methodological implications. Clinically, TUS demonstrates favorable compatibility and additive efficacy when combined with diverse rehabilitation modalities, supporting its broad applicability as a “rehabilitation booster.” Methodologically, however, we cannot precisely quantify the isolated therapeutic contribution of TUS nor confirm consistent efficacy across distinct rehabilitation backgrounds. Rigorous three-arm randomized controlled trials or dose-response studies implemented under unified standardized rehabilitation protocols are urgently needed in future research to disentangle the independent and additive effects of TUS.

### 4.3 Parameter variation and individualized strategies

No unified standardized parameter guidelines for TUS have been established for stroke rehabilitation to date. The included literature revealed that 800 kHz was the most widely adopted ultrasound frequency. Stimulation intensities predominantly ranged from 0.54 to 1.75 W/cm^2^, with 0.75 W/cm^2^ representing the most commonly used setting that balances therapeutic benefit and safety. Single-session treatment durations mostly fell between 20 and 30 min, with the temporal window serving as the primary stimulation target; multi-target protocols combining the temporal window with lesion and vascular projection zones were also frequently reported. Nevertheless, TUS parameters varied considerably across trials, and most publications failed to fully document pulse-related metrics, which impairs cross-study comparability and reproducibility and underscores the requirement for complete, standardized parameter reporting in future clinical trials.

Exploratory subgroup analyses stratified by TUS technical parameters preliminarily delineated parameter-response associations for activities of daily living (BI) and motor function (FMA). For BI outcomes, subgroups adopting low frequency (<800 kHz) and low intensity (≤ 0.75 W/cm^2^) produced larger effect sizes. This advantage was largely driven by a single trial (Zhao et al., 2025) utilizing a 700 kHz frequency, 0.65 W/cm^2^ intensity, a 4-week treatment course, and participants with severe baseline functional dependence. The observed inter-subgroup differences were therefore more attributable to patient characteristics and treatment duration than frequency or intensity per se. No clear linear dose-response gradient was detected within the clinically prevalent intensity range of 0.65–1.75 W/cm^2^, indicating stimulation intensity is not the primary determinant of therapeutic response. After removing outlier data, multi-target stimulation protocols yielded effect sizes comparable to those of unilateral temporal window stimulation, confirming that isolated temporal window targeting is sufficient to achieve meaningful functional improvements.

For FMA motor outcomes, comparable therapeutic effects and low within-subgroup heterogeneity were observed between the < 800 and 800 kHz strata; the widely utilized 800 kHz frequency yielded stable motor recovery benefits. Only one trial adopted frequencies exceeding 800 kHz and reported smaller effect estimates, a finding that should not be overinterpreted given the limited sample size of this stratum.

Notably, the parameter subgroup analyses were constrained by limitations inherent to the available dataset. Several subgroups included only one or two trials, and most manuscripts lacked full documentation of critical pulse metrics, such as duty cycle and pulse repetition frequency. Unresolvable heterogeneity precluded duration-stratified analyses for both BI and FMA, as well as intensity- and site-stratified analyses for FMA. This highlights the urgent need for uniform, comprehensive parameter reporting in subsequent TUS trials to enable robust dose-response quantitative synthesis.

### 4.4 Differential effects across functional domains

Subgroup analyses demonstrated that TUS produced significantly greater cognitive improvements in patients in the non-acute stroke phase (> 14 days post-onset) than in acute counterparts. This aligns with the temporal neuroplasticity window theory: the subacute and chronic phases constitute a critical period for synaptic remodeling and neural network reorganization. During this window, TUS exerts catalytic restorative effects through multiple mechanisms, including suppressing neuroinflammation by shifting microglia toward the anti-inflammatory M2 phenotype, upregulating BDNF to facilitate dendritic spine formation, and optimizing intracranial microcirculation by enhancing cerebrospinal fluid circulation (Choi et al., 2025; Ikezoe et al., 2018; Qin et al., 2025). Clinically, TUS combined with targeted cognitive training is recommended for patients with residual cognitive impairment beyond the acute phase to capitalize on the neuroplastic golden period.

In terms of Barthel Index (BI) outcomes measuring daily living performance, patients with severe baseline functional dependence (BI ≤ 40) exhibited larger absolute functional gains following TUS intervention. This suggests TUS may modulate homeostatic plasticity (Ding et al., 2024) to help patients break through conventional rehabilitation plateaus, acting as a “neural primer” or enhancer. For individuals with stagnant functional progress after standard rehabilitation, adjunct TUS is proposed to reactivate neural circuit responsiveness to subsequent training.

Regarding swallowing dysfunction, only two eligible RCTs were incorporated in this meta-analysis. Although a favorable therapeutic trend was detected, the overall evidence strength remains insufficient. Swallowing regulation relies on a sophisticated multi-layered network encompassing the brainstem, cerebral cortex, and basal ganglia (Stampanoni Bassi et al., 2025), which demands more precise targeted stimulation and extended intervention courses. Future research should prioritize high-quality trials investigating TUS for post-stroke dysphagia, an understudied yet clinically relevant functional impairment.

### 4.5 Limitations

The present meta-analysis also has limitations: ➀Biases in literature retrieval and inclusion: This study only searched mainstream Chinese and English databases, without systematic screening of grey literature, non-Chinese/English publications, or unpublished trial data. Retrieval bias may compromise the objectivity and comprehensiveness of pooled effect estimates. ➁Geographical and population generalizability: The majority of included studies originated from a single linguistic and regional context. International multi-center RCTs are required to validate whether these conclusions extend to diverse ethnic cohorts and healthcare systems. ➂Publication bias assessment: Egger’s regression test was only feasible for the Barthel Index (*n* = 11, *P* > 0.05) due to adequate sample size. Formal publication bias testing could not be performed for cognitive, motor, and swallowing endpoints owing to limited trial numbers, raising the risk of overestimated treatment effects in these domains. ➃Constraints of parameter subgroup analyses: Stratified analyses by frequency, intensity, and stimulation site were only viable for BI, while merely frequency stratification was available for FMA. Other parameter-based subgroup comparisons could not be implemented due to excessive heterogeneity or insufficient trials, thereby prohibiting the identification of definitive optimal TUS threshold values. ➄Core limitation: Ambiguous attribution of therapeutic effects. As described above, baseline rehabilitation regimens varied drastically across included trials. The pooled effect sizes herein should be interpreted as composite gains from TUS added to heterogeneous, individualized rehabilitation frameworks, rather than the standardized, isolated efficacy of TUS monotherapy. This limitation substantially reduces confidence in the direct extrapolation of effect magnitudes to routine clinical practice.

## 5 Conclusion

In summary, current evidence indicates that adding TUS to conventional rehabilitation effectively promotes the recovery of neurological, motor, cognitive, swallowing, and daily living function in stroke patients, with particular benefits observed in individuals with moderate to severe baseline disability. Clinical TUS applications should follow individualized principles, with treatment timing and combined regimens tailored to the stroke phase and predominant functional deficits. Future research directions are outlined as follows: ➀Conduct large-scale, multi-center, rigorously designed RCTs (especially three-arm trials) to disentangle the independent and additive therapeutic values of TUS; ➁Establish standardized reporting protocols for all TUS technical parameters in line with statements such as TRIPOD, and construct a comprehensive parameter-response database; ➂Integrate neuroimaging and electrophysiological modalities to elucidate acute and sustained neuromodulatory mechanisms of TUS in the human brain; ➃Launch dedicated RCTs focusing on understudied functional domains including dysphagia and aphasia. Ultimately, more rigorous trial design and translational clinical research will advance precise, standardized implementation of transcranial ultrasound stimulation within stroke rehabilitation workflows.

## Data Availability

The original contributions presented in the study are included in the article/Supplementary material, further inquiries can be directed to the corresponding authors.
