# Supplementary material for: Transcranial ultrasound stimulation for post-stroke dysfunction: a meta-analysis of randomized controlled trials
Source: Front Neurosci. 2026 Aug 7;20:1828888. doi: 10.3389/fnins.2026.1828888 (PMC13493570; doi:10.3389/fnins.2026.1828888)
Supplement: Supplementary file 1 [file Data_Sheet_1.docx]

Supplementary Material

1 **Search strategy for Cochrane Library**

Pubmed :

("Stroke"[Mesh] OR (Strokes[Title/Abstract] OR "Cerebrovascular Accident"[Title/Abstract] OR "Cerebrovascular Accidents"[Title/Abstract] OR "Cerebral Stroke"[Title/Abstract] OR "Cerebral Strokes"[Title/Abstract] OR "Stroke, Cerebral"[Title/Abstract] OR "Strokes, Cerebral"[Title/Abstract] OR "Cerebrovascular Apoplexy"[Title/Abstract] OR "Apoplexy, Cerebrovascular"[Title/Abstract] OR "Vascular Accident, Brain"[Title/Abstract] OR "Brain Vascular Accident"[Title/Abstract] OR "Brain Vascular Accidents"[Title/Abstract] OR "Vascular Accidents, Brain"[Title/Abstract] OR "Cerebrovascular Stroke"[Title/Abstract] OR "Cerebrovascular Strokes"[Title/Abstract]) AND ("Transcranial Ultrasound" [Title/Abstract] OR "Transcranial Ultrasonic Therapy" [Title/Abstract] OR "Low-Intensity Pulsed Ultrasound"[Title/Abstract] OR LIPUS OR "Therapeutic Ultrasound" [Title/Abstract] OR "Focused Ultrasound" [Title/Abstract] OR "Pulsed Ultrasound"[Title/Abstract] )

Web of Science:

(TS=(Strok* OR "Cerebrovascular Accident*" OR "Cerebral Stroke" OR "Cerebrovascular Apoplexy" OR "Brain Vascular Accident*")) AND (TS=("Transcranial Ultrasound" OR "Transcranial Ultrasonic Therapy" OR "Low-Intensity Pulsed Ultrasound" OR LIPUS OR "Therapeutic Ultrasound" OR "Focused Ultrasound" OR "Pulsed Ultrasound"))

Cochrane Library:

#1:MeSH descriptor: [Stroke]

#2: (Strokes, Cerebrovascular):ti, ab,kw OR (CVA (Cerebrovascular Accident)):ti, ab,kw OR (Cerebrovascular Strokes):ti,ab,kw OR (Cerebrovascular Accident):ti, ab, kw OR (Brain Vascular Accidents):ti, ab,kw OR (Cerebrovascular Accidents):ti, ab, kw OR (Cerebral Strokes):ti, ab,kw OR (Cerebral Stroke):ti, ab, kw OR (CVAs (Cerebrovascular Accident)):ti, ab, kw OR (Stroke, Cerebral):ti,ab, kw OR (Vascular Accidents, Brain):ti,ab, kw OR (Cerebrovascular Apoplexy):ti, ab, kw OR (Apoplexy, Cerebrovascular):ti, ab,kw OR (Brain Vascular Accident):ti, ab, kw OR (Strokes, Cerebral):ti, ab, kw OR (Vascular Accident, Brain):ti, ab, kw OR (Apoplexy):ti, ab, kw OR (Cerebrovascular Stroke):ti, ab, kw OR (Stroke, Cerebrovascular):ti, ab, kw OR (Strokes):ti, ab, kw OR (Acute Stroke):ti, ab, kw OR (Cerebrovascular Accidents, Acute):ti, ab, kw OR (Acute Cerebrovascular Accidents):ti, ab, kw OR (Acute Cerebrovascular Accident):ti, ab, kw OR (Cerebrovascular Accident, Acute):ti, ab, kw

#3: #1 OR #2

#4: (Transcranial Ultrasound):ti, ab,kw OR (Transcranial Ultrasonic Therapy):ti, ab, kw OR (Low-Intensity Pulsed Ultrasound):ti, ab, kw OR (LIPUS):ti, ab, kw OR (Therapeutic Ultrasound):ti, ab, kw OR (Focused Ultrasound):ti, ab, kw OR (Pulsed Ultrasound):ti,ab, kw

#5: Final search :#3 AND #4

Embase:

#1: 'cerebrovascular accident'/exp

#2: 'accident, cerebrovascular':ab,ti OR 'acute cerebrovascular lesion':ab,ti OR 'acute focal cerebral vasculopathy':ab,ti OR 'acute stroke':ab,ti OR 'apoplectic stroke':ab,ti OR 'apoplexia':ab,ti OR 'apoplexy':ab,ti OR 'blood flow disturbance, brain':ab,ti OR 'brain accident':ab,ti OR 'brain infarction':ab,ti OR 'brain blood flow disturbance':ab,ti OR 'brain insult':ab,ti OR 'brain insults':ab,ti OR 'brain vascular accident':ab,ti OR 'cerebral apoplexia':ab,ti OR 'cerebral insult':ab,ti OR 'cerebral stroke':ab,ti OR 'cerebral vascular accident':ab,ti OR 'cerebral vascular insufficiency':ab,ti OR 'cerebro vascular accident':ab,ti OR 'cerebrovascular arrest':ab,ti OR 'cerebrovascular failure':ab,ti OR 'cerebrovascular injury':ab,ti OR 'cerebrovascular insufficiency':ab,ti OR 'cerebrovascular insult':ab,ti OR 'cerebrum vascular accident':ab,ti OR 'cryptogenic stroke':ab,ti OR 'cva':ab,ti OR 'insults cerebrials':ab,ti OR 'ischaemic seizure':ab,ti OR 'ischemic seizure':ab,ti OR 'thrombotic stroke':ab,ti

#3: #1 OR #2

#4: 'focused ultrasound therapy'/exp

#5: 'focused ultrasonic ablation':ab,ti OR 'focused ultrasonic irradiation':ab,ti OR 'focused ultrasound':ab,ti OR 'focused ultrasound treatment':ab,ti OR 'focused ultrasound therapy':ab,ti

#6: #4 OR #5

#7: Final search: #3 AND #6

CNKI：

（经颅超声刺激 + 经颅超声+ 颅脑超声刺激 + 脑超声神经调控 + TUS） AND （脑卒中 + 中风 + 脑梗死 + 脑梗塞 + 脑出血）

Wanfang Medical Network：

经颅超声刺激 OR 经颅超声 OR 低强度聚焦超声 OR 经颅脉冲超声 OR 颅脑超声刺激 OR TUS) AND（脑卒中 OR 中风 OR 脑梗死 OR 脑出血 OR 缺血性卒中 OR 出血性卒中 OR 卒中后功能障碍）
